# Cathepsin S Is Involved in Th17 Differentiation Through the Upregulation of IL-6 by Activating PAR-2 after Systemic Exposure to Lipopolysaccharide from *Porphyromonas gingivalis*

**DOI:** 10.3389/fphar.2017.00470

**Published:** 2017-07-17

**Authors:** Masato Dekita, Zhou Wu, Junjun Ni, Xinwen Zhang, Yicong Liu, Xu Yan, Hiroshi Nakanishi, Ichiro Takahashi

**Affiliations:** ^1^Section of Orthodontics and Dentofacial Orthopedics, Kyushu University Fukuoka, Japan; ^2^Department of Aging Science and Pharmacology, Kyushu University Fukuoka, Japan; ^3^OBT Research Center, Faculty of Dental Science, Kyushu University Fukuoka, Japan; ^4^Center of Implant Dentistry, School of Stomatology, China Medical University Shenyang, China; ^5^The VIP Department, School of Stomatology, China Medical University Shenyang, China

**Keywords:** lipopolysaccharide derived from *Porphyromonas gingivalis*, Cathepsin S, dendritic cells, IL-6, Th17 cells

## Abstract

Positive links have been found between periodontitis and numerous diseases in humans via persistent inflammation throughout the body. However, the main factors responsible for maintaining this pro-inflammatory condition are poorly understood. The spleen, the largest secondary immune organ, is a central hub regulating the immune response/inflammation due to the dendritic cell (DC) response to CD4^+^ T cell subtype differentiation, and lysosomal proteinase cathepsin S (CatS) is known to be involved in DC functions. In the present study, we found that CatS-induced IL-6 production by splenic DCs subsequently promotes Th17 differentiation, in response to systemic exposure to lipopolysaccharide derived from *Porphyromonas gingivalis* (PgLPS). The population of CD11c^+^ DCs was significantly increased in the splenic marginal zone (MZ) locally of wild-type (DBA/2) mice with splenomegaly but not in that of CatS deficient (*CatS^-/-^*) mice after systemic exposure to PgLPS for 7 consecutive days (5 mg/kg/day, intraperitoneal). Similarly, the population of Th17^+^CD4^+^ T cells was also significantly increased in the splenic MZ of wild-type mice but not in that of *CatS^-/-^* mice after PgLPS exposure. Furthermore, the increase in the Th17^+^ CD4^+^ T cell population paralleled increases in the levels of CatS and IL-6 in CD11c^+^ cells in the splenic MZ. In isolated primary splenic CD11c^+^ cells, the mRNA expression and the production of IL-6 was dramatically increased in wild-type mice but not in *CatS^-^*^/-^ mice after direct stimulation with PgLPS (1 μg/ml), and this PgLPS-induced increase in the IL-6 expression was completely abolished by pre-treatment with Z-Phe-Leu-COCHO (Z-FL), the specific inhibitor of CatS. The PgLPS activated protease-activated receptor (PAR) 2 in the isolated splenic CD11c^+^ cells was also significantly inhibited by *CatS* deficiently. In addition, the PgLPS*-*induced increase in the IL-6 production by splenic CD11c^+^ cells was completely abolished by pre-treatment with FSLLRY-NH_2_, a PAR2 antagonist, as well as Akti, a specific inhibitor of Akt. These findings indicate that CatS plays a critical role in driving splenic DC-dependent Th17 differentiation through the upregulation of IL-6 by activating PAR2 after exposure to components of periodontal bacteria. Therefore, CatS-specific inhibitors may be effective in alleviating periodontitis-related immune/inflammation.

## Introduction

Periodontitis, a common oral chronic multi-bacterial infection, results from the relevant bacteria and their components entering the body through the bloodstream. *Porphyromonas gingivalis (P. gingivalis*) is the major periodontal bacteria (Seymour et al., 2007), and its lipopolysaccharide derived from *P. gingivalis* (PgLPS) induces executive pro-inflammatory responses through various types of cells, including macrophages, leptomeningeal cells, microglia and fibroblasts (Wu et al., 2008; Liu et al., 2013; Wu and Nakanishi, 2015; Li et al., 2016). Recently, substantial clinical evidence has shown that periodontitis causes or hastens not only other systemic diseases, such as atherosclerosis and diabetes (Lalla and Papapanou, 2011; Velsko et al., 2014; Esteves Lima et al., 2016) and rheumatoid arthritis (Leech and Bartold, 2015; Silvestre et al., 2016), but also neurodegenerative diseases, such as Alzheimer’s disease (Noble and Scarmeas, 2009; Poole et al., 2013), through persistent systemic inflammation and neuroinflammation. However, the factors responsible for the maintenance of this whole-body pro-inflammatory condition are poorly understood.

The spleen is the largest secondary immune organ (SLO) for regulating the immune response and inflammation (Lind and Lind, 2012; Zhao et al., 2015) and is particularly susceptible to infection because of its anatomical structures (Cadili and de Gara, 2008; Getts et al., 2011; Tarantino et al., 2011). The marginal zones (MZs), in which abundant dendritic cells (DCs) and macrophages interface with T-cell zones (Bajénoff et al., 2008; Ruddle and Akirav, 2009), are considered therapeutic targets for modulating innate and adaptive immunity (Zhao et al., 2015).

Cathepsin S (CatS; EC 3.4.22.27) is a lysosomal cysteine protease preferentially expressed in phagocytic cells (Petanceska et al., 1996). It has a broad pH profile and can be active at a neutral pH, consistent with its extracellular biological activity (Storm van’s Gravesande et al., 2002). CatS mainly controls MHC class II antigen-presenting cells, including DCs (Driessen et al., 1999; Nakagawa and Rudensky, 1999; Shi et al., 1999; Riese et al., 2001; Nakanishi, 2003), and we recently found that CatS is involved in the regulation of splenic CD4^+^ T-cell-dependent responses (Zhang et al., 2014).

We hypothesized that the enzymatic activity of CatS is required for the persistence/exacerbation of the splenic immune response/inflammation through DC-dependent CD4^+^T cell activation and subtype differentiation during periodontitis infection. To confirm our hypothesis, we examined the effects of a genetic CatS deficiency and a specific CatS inhibitor on the CD4^+^ T-cell activation and subset differentiation after systemic exposure to PgLPS.

## Materials and Methods

### Reagents

PgLPS was purchased from InvivoGen (San Diego, CA, United States). Akt inhibitor (Akti) and Z-Phe-Leu-COCHO (Z-FL, a specific inhibitor of CatS) were purchased from Calbiochem (CA, United States). Phe-Ser-Leu-Leu-Arg-Try-NH_2_ (FSLLRY-NH_2_, an antagonist peptide of PAR2) was purchased from SIGMA-ALDRICH (St. Louis, United States).

### Animals

The study was carried out in accordance with the recommendations of the Institutional Animal Care and Use Committee of Kyushu University. The protocols was approved by the Institutional Care and Use Committee of Kyushu University. Eight- to 10-week-old female wild-type and CatS-deficient (*CatS^-/-^*) mice on a DBA/2 background were used for the experiments. Genotyping was performed as described previously (Hao et al., 2007). The mice were maintained on a 12-h light/dark cycle (lights on at 8:00 A.M.) at 22–25°C ambient temperature with food and water available ad libitum. All of the mice were handled daily for 5 days before the start of the experiment to minimize their stress reactions to manipulation. To induce a systemic immune response/inflammation, mice were injected intraperitoneally with either salineor PgLPS (a TLR2 agonist, 5 mg/kg, i.p.), the same dose at which other TLR2 agonists have been used in mice (Eklind et al., 2004; Du et al., 2011).

### Locomotor Activity

The spontaneous locomotor activity was measured in a clean, novel cage similar to the home cage, devoid of bedding or litter between 1:00 and 3:00 p.m. The cage was divide into four virtual quadrants. Wild-type (*n* = 6) and *CatS^-/-^* (*n* = 6) mice were kept in the cage. The locomotor motor activity was measured by counting the number of line crossings and rearing incidents over a 5-min period.

### Hematoxylin and Eosin Staining

Mice were sacrificed, and the spleen tissues were fixed in 4% paraformaldehyde for 24 h and then washed with phosphate-buffered saline (PBS) and embedded in paraffin. The samples of spleen for hematoxylin-eosin (HE) staining were prepared as described previously (Wu et al., 2000). The specimens were cryoprotected at 4°C for 2 days in 30% sucrose in PBS and then embedded in an optimal cutting temperature compound (Sakura Finetechnical Co., Ltd., Tokyo, Japan). Serial coronal frozen sections (thickness: 14 μm) of the spleen were subjected to the immunohistochemical analyses.

### Double-Immunofluorescent Staining

The samples of the spleens from wild-type and *CatS^-/-^* mice were obtained 7 days after the LPS or saline injection. The spleen sample sections were incubated with the following antibodies for 2 days at 4°C: rat anti-CD4 (1:500; BD PharMingen, New Jersey, USA) rabbit anti-IFNγ (1:1000; Life Technologies, CA, United States), goat anti-CatS (M-19, 1:500; Santa Cruz Biotechnology, CA, United States) rabbit anti-IL-17 (1:500; Santa Cruz Biotechnology), goat anti-IL-4 (1:500; Santa Cruz Biotechnology), rabbit anti-TGFβ (1:500; Santa Cruz Biotechnology) and mouse anti-TLR2 (1:500; eBioscience, San Diego, CA, United States). The sections were then washed with PBS and incubated with a mixture of secondary antibodies conjugated with donkey anti-rabbit AlexaFluor 488 (1:400; Jackson ImmunoResearch, PA, United States) and donkey anti-rat Cy3 (1:400; Jackson ImmunoResearch), donkey anti-goat AlexaFluor 488 (1:400; Jackson ImmunoResearch) and donkey anti-rabbit Cy3 (1:400; Jackson ImmunoResearch) or donkey anti-mouse Cy3 (1:500; Jackson ImmunoResearch), donkey anti-goat AlexaFluor 488 (1:400), and donkey anti-rabbit Cy3 (1:400), or donkey anti-mouse Cy3 (1:500) for 3 h at 24°C. The spleen sample sections were then incubated with the following antibodies: rat anti-CD4 (1:500 BD PharMingen, New Jersey, United States), as described previously (Ni et al., 2015). In the immune-stained images, the cell number was determined by manually examination, and the percentage of cells was calculated by dividing the number of co-stained cells by the number of single-stained CD4^+^ or CD11c^+^ cells.

### Western Blotting

Mice were transcardially perfused with PBS, and then the spleen was removed. The specimens were quickly frozen and stored at -80°C. Each specimen was electrophoresed using 15 or 12% SDS-polyacrylamide gels. The proteins on SDS gels were transferred electrophoretically to nitrocellulose membranes. The membranes were washed with PBS and incubated at 4°C overnight under gentle agitation with each primary antibody: goat anti-CatS (M-19, 1:1000; Santa Cruz Biotechnology), goat anti-cleaved IL-6 (1:500; Santa Cruz Biotechnology), or mouse anti-actin (1:5000; Abcam, Cambridge, United Kingdom). After being washed, the membranes were incubated with horseradish peroxidase (HRP)-anti-goat (1:2000 R&D Systems, Minneapolis, USA) or anti-mouse (1:2000; GE Healthcare, United Kingdom) antibodies for 2 h at room temperature. Subsequently, the membrane-bound, HRP-labeled antibodies were detected using an enhanced chemiluminescence detection system (ECK lit; GE Healthcare) with an image analyzer (LAS-3000; Fuji Photo Film, Tokyo, Japan).

### Cell Isolation and Cultures

Splenocytes, CD4^+^ cells and CD11c^+^ cells were isolated from the mouse spleens. The CD11c^+^ cells could be inducted as splenic DCs recently (Owens et al., 2012; Bao et al., 2015; Wang et al., 2017). Three mice in each group were anesthetized and perfused transcardially with PBS, and then the spleen samples were cut into small pieces. After enzymatic digestion using the Neural Tissue Dissociation Kit (Papain, Bergisch Gladbach, Germany), the cell suspensions were further mechanically dissociated using a gentle MACS Dissociator (Milteny Biotec, Bergisch Gladbach, Germany), and single splenocyte suspensions were obtained after the samples were applied to a 30-μm cell strainer. After removing the red blood cells using Red Blood Cell Lysis Solution (Miltenyi Biotec), the CD11c^+^ cells were magnetically labeled with CD11c MicroBeads, and the CD4^+^ cells were labeled with CD4 MicroBeads. The cell suspension was then loaded onto a MACS column placed in the magnetic field of a MACS separator, and the CD11c^+^ or CD4^+^ cells were eluted after removing the magnetic field in accordance with the previously described methods (Wu et al., 2013). The cultured media of the isolated CD11c^+^ cells were collected 24 h after exposure to PgLPS for 24 h (1 μg/ml). In the co-culture experiments, CD11c^+^ cells were co-cultured with CD4^+^ cells after isolation. The CD4^+^ cells were seeded into 6-well plates, and CD11c^+^ cells were cultured in separate inserts (CORNING) separated by membranes with 0.4-μm pores. The free flux of cytokines was then allowed between the two types of cells. The cells and the cultured medium were collected 24 h after PgLPS (1 μg/ml) treatment.

### Assay for IL-6 by ELISA

The collected CD11c^+^ cell-cultured media and the co-cultured media were measured using an IL-6 ELISA kit (R&D) in accordance with the manufacturer’s protocol. The absorbency at 450 nm was measured using a microplate reader.

### Quantitative Real-Time Reverse Transcription Polymerase Chain Reaction Analysis

To examine the expression of inflammatory-related molecules by DCs and their effects on CD4^+^ T cells after exposure to Pg LPS, total RNA was extracted from DCs or CD4^+^ T cells at different time points after exposure to Pg LPS using RNAiso (Takara Bio Inc., Shiga, Japan). Complementary DNA (cDNA) was prepared via reverse transcription of the total RNA using a QuantiTect Reverse Transcription Kit (QIAGEN, Hilden, Germany). The diluted cDNA samples were analyzed via real-time reverse transcription polymerase chain reaction (RT-PCR). Real-time PCR was performed using the Rotor-Gene SYBR Green RT-PCR Kit (QIAGEN) and Corbett Rotor-Gene RG-3000A Real-Time PCR System (Biocompare, Sydney, Australia). We determined the copy numbers via the 2-Ct method using a calibrator. The sequences of the primer pairs are as follows: IL-6: 5′-TCAATTCCAGAAACCGCTATGA-3′ and 5′-CACCAGCATCAGTCCCA AGA-3′; Rorc: 5′-GCCTCCTGCCACCTTGAGT-3′ and 5′-TCTGCCTTCAGCTTTGCCTC-3′; Tbx21: 5′-CACTAAGCAAGGACGGCGAA-3′ and 5′-CCACCAAGACCACATCCACA-3′; Foxp3: 5′-GAAGCTGGGAGCTATGCAGG-3′ and 5′-TGGCTACGATGCAGCAAGAG-3′; GATA3: 5′-TTTACCCTCCGGCTTCATCCTCCT-3′ and 5′-TGCACCTGATACTTGAGGCACTCT-3′; IL-17: 5′-ACCTCAACCGTTCCACGTCA-3′ and 5′-CAGGGTCTTCATTGCGGTG-3′; Actin: 5′-AGAGGGAAATCGTGCGTGAC-3′ and 5′-CAATAGTGATGACCTGGCCGT-3 ′. For the data normalization, all gene expressions were normalized to actin.

### Statistical Analyses

All of the data are shown as the mean ± SEM. The statistical analyses were performed using a two-tailed unpaired Student’s *t*-test, a one-way analysis of variance (ANOVA) with a *post hoc* Tukey’s test or a two-way ANOVA with repeated measurements using the GraphPad Prism 7 software package (GraphPad Software Inc., San Diego, CA, United States). Unless otherwise indicated, the data met the assumptions of equal variances. The differences were considered to be significant at *p* < 0.05.

## Results

### CatS IS Involved in Splenic CD11c^+^ DC Activation after Systemic Exposure to PgLPS

**FIGURE 1:**
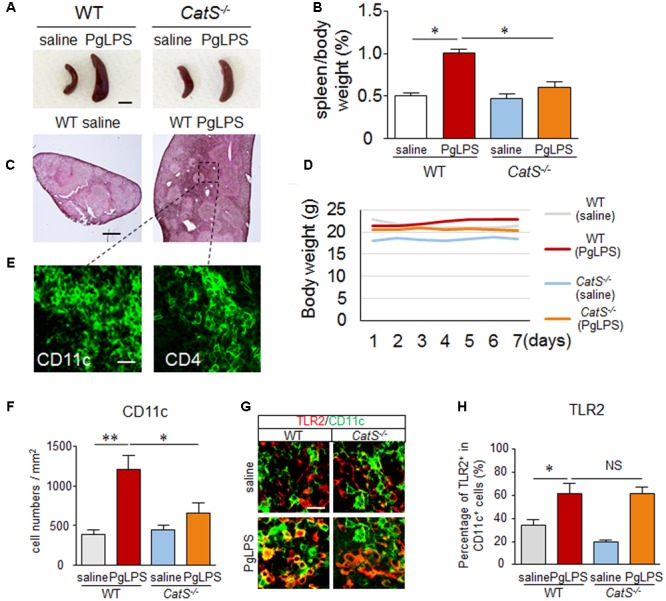
CatS is involved in splenic CD11c^+^ DC activation after systemic exposure to PgLPS. **(A)** The suppression of the systemic exposure to PgLPS-induced splenic hypertrophy by the genetic deletion of CatS. Scale bar, 0.5 cm. **(B)** The mean spleen weight per body weight in both wild-type and *CatS^-/-^* mice at 7 days after systemic exposure to PgLPS. Each column and vertical bar represent the mean ± SEM of six independent experiments (*n* = 6). Asterisks indicate a statistically significant difference between the values (^∗^*p* < 0.05). **(C)** The HE staining of wild-type mice spleen at 7 days after exposure to PgLPS. The pane indicates the marginal zone (MZ), which will be studied in the subsequent experiments. Scale bar, 200 μm. **(D)** The body weight in both wild-type (*n* = 6) and *CatS^-/-^* mice (*n* = 6) after systemic exposure to PgLPS. **(E)** The immunofluorescent CLSM images of CD11c and CD4 in wild-type mice spleen 7 days after exposure to PgLPS. Scale bar, 15 μm. **(F)** The quantitative analyses of CD11c-positive cells in the MZ of mice at 7 days after exposure to PgLPS. Each column and vertical bar represent the mean ± SEM of six independent experiments (*n* = 6). Asterisks indicate a statistically significant difference between the values (^∗^*p* < 0.05, ^∗∗^*p* < 0.01). **(G)** The immunofluorescent CLSM images of CD11c (green) and TLR2 (red) in the MZ of mice at 7 days after exposure to PgLPS. Scale bar, 20 μm. **(H)** The mean ratio of TLR2^+^ cells in the total CD11c^+^ cells in the MZ of mice at 7 days after exposure to PgLPS or saline. Each column and vertical bar represent the mean ± SEM of six independent experiments (*n* = 6). Asterisks indicate a statistically significant difference between the values (^∗^*p* < 0.05). NS, no significant difference.

First, we examined the involvement of CatS in the response of the spleen after systemic exposure to PgLPS, a TLR2 agonist, as the spleen is susceptible to infection. The spleen size was significantly greater in the PgLPS-exposed wild-type mice than in the endotoxin-free saline-treated mice, as was the relative spleen weight-to-body weight ratio at day 7 after systemic exposure to PgLPS (**Figures 1A,B**). Surprisingly, PgLPS-induced splenomegaly was markedly suppressed in the *CatS^-/-^* mice (**Figures 1A,B**). A histological analysis showed a dramatic increase in the population of splenocytes in the MZ of the PgLPS-exposed wild-type mice (**Figure 1C**) but not in that of the *CatS^-/-^* mice (data not shown). Those observations indicate that the systemic PgLPS-induced dynamic responses in the splenic MZ were prevented by CatS deficiency. Sickness behaviors-related symptoms such as a reduction in the body weight (**Figure 1D**) or lethargy (data not shown) were not observed even expose to the high dose of PgLPS (5 mg/kg). The significant increase in the number of CD11c^+^ DCs (threefold increase) paralleled the increase in the TLR2 expression in CD11c^+^ DCs (twofold increase) in the splenic MZ of wild-type mice after systemic exposure to PgLPS, indicating that DCs in the splenic MZ sensitively respond to TLR2 activation (**Figures 1E–H**). We noted that the number of CD11c^+^ DCs in the splenic MZ, but not the TLR2 expression in the CD11C ^+^ DCs, was significantly reduced in *CatS^-/-^* mice compared with wild-type mice (**Figures 1E–H**), indicating that CatS is involved in splenic CD11c^+^ DC functions downstream of TLR2 activation.

### CatS IS Involved in Splenic Th17^+^ Cell Differentiation during Systemic Exposure to PgLPS

**FIGURE 2:**
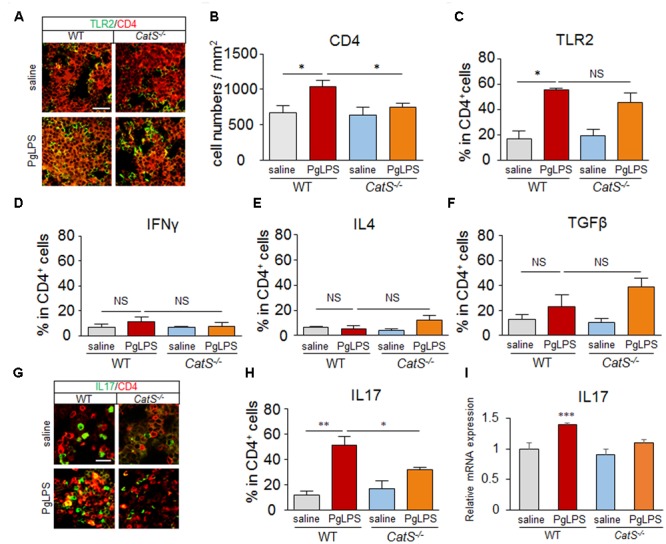
CatS is involved in splenic Th17^+^ cell differentiation after systemic exposure to PgLPS. **(A)** The immunofluorescent CLSM images of CD4 (red) and TLR2 (green) in the MZ of mice at 7 days after exposure to PgLPS. Scale bar, 40 μm. **(B)** The quantitative analyses of CD4-positive cells in the MZ of the mice at 7 days after exposure of PgLPS. Each column and vertical bar represent the mean ± SEM of six independent experiments (*n* = 6). Asterisks indicate a statistically significant difference between the value (^∗^*p* < 0.05). **(C–F)** The mean ratio of TLR2^+^, IFNγ^+^, IL4^+^, TGFβ^+^ cells, respectively, in the total CD4^+^ cells in the MZ of mice at 7 days after exposure to PgLPS or saline. Each column and vertical bar represent the mean ± SEM of six independent experiments (*n* = 6). Asterisks indicate a statistically significant difference between the values (^∗^*p* < 0.05, ^∗∗^*p* < 0.01). NS, no significant difference. **(G)** The immunofluorescent CLSM images of CD4 (red) and IL17 (green) in the MZ of mice at 7 days after exposure to PgLPS. Scale bar, 20 μm. **(H)** The mean ratio of IL17^+^ cells in the total CD4^+^ cells in the MZ of mice at 7 days after exposure to PgLPS or saline. Each column and vertical bar represent the mean ± SEM of six independent experiments (*n* = 6). Asterisks indicate a statistically significant difference between the values (^∗^*p* < 0.05, ^∗∗^*p* < 0.01). **(I)** The mRNA expression of IL17 in the spleens of mice at 7 days after exposure to PgLPS. Each column and vertical bar represent the mean ± SEM of six independent experiments (*n* = 6). Asterisks indicate a statistically significant difference between the values (^∗∗∗^*p* < 0.001).

Next, we examined the involvement of CatS in the subtype of CD4^+^ T cells in the splenic MZ after systemic exposure to PgLPS, as CD4^+^ T cells are closely interfaced to CD11c^+^ DCs anatomically in the splenic MZ. The significant increase in the population of CD4^+^ T cells paralleled the increase in their TLR2 expression near the splenic MZ of wild-type mice after systemic exposure to PgLPS, and the number of CD4^+^ T cells but not their TLR2 expression was significantly reduced in *CatS^-/-^* mice compared with wild-type mice (**Figures 2A–C**), suggesting that CatS is involved in regulating splenic CD4^+^ T cells downstream of TLR2 activation.

The mean percentages of IFNγ^+^ and IL-4^+^ in CD4^+^ T cells were not increased (**Figures 2D,E**), and that of TGFβ^+^ was not significantly increased (**Figure 2F**) in wild-type or *CatS^-/-^* mice, however, the mean percentage of IL-17^+^ in CD4^+^ T cells was dramatically increased (4.5-fold) near the splenic MZ after systemic exposure to PgLPS. Surprisingly, the mean percentage of IL-17^+^ in CD4^+^ T cells was significantly lower in the *CatS^-/-^* mice than in the wild-type mice (**Figures 2G,H**). Furthermore, the mean mRNA level of IL17 in the spleen of PgLPS-exposed wild-type mice was significantly higher than in the saline-exposed mice, but not significantly higher than in the Pg-LPS-exposed *CatS^-/-^* mice (**Figure 2I**). These observations strongly suggest that CatS is involved in the differentiation of splenic Th17^+^ cells, which agrees a previous study showing that the IL-17 cells induced by *P. gingivalis* are depended on TLR2 activation on DCs (de Aquino et al., 2014).

### CatS IS Essential for the Production of IL-6 by Splenic CD11c^+^ DCs after Systemic Exposure to PgLPS

**FIGURE 3:**
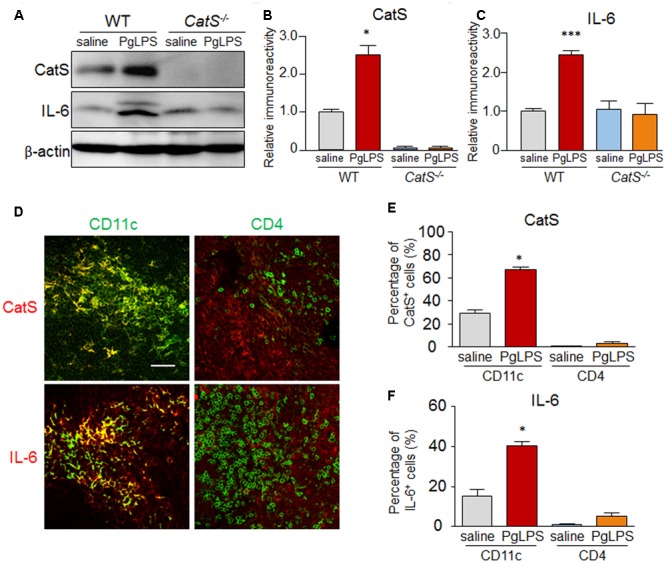
CatS is involved in IL-6 production by splenic CD11c^+^ DCs after systemic exposure to PgLPS. **(A)** The immunoblots show the levels of CatS and IL-6 in the spleens of mice at 7 days after exposure to PgLPS. **(B,C)** The quantitative analyses of CatS **(B)** and IL-6 **(C)** in the immunoblots shown in **(A)**. Each column and vertical bar represent the mean ± SEM of six independent experiments (*n* = 6). Asterisks indicate a statistically significant difference between the values (^∗^*p* < 0.05, ^∗∗∗^*p* < 0.001). **(D)** The immunofluorescent CLSM images of CatS (red) and IL-6 (red) in the CD11c^+^ and CD4^+^ cells in the MZ of wild-type mice spleens at 7 days after exposure to PgLPS. Scale bar, 30 μm. **(E,F)** The mean ratio of CatS^+^ **(E)** and IL-6^+^ **(F)** cells in the total CD4^+^ and CD11c^+^ cells in the MZ of the mice at 7 days after exposure to PgLPS or saline. Each column and vertical bar represent the mean ± SEM of six independent experiments (*n* = 6). Asterisks indicate a statistically significant difference between the values (^∗^*p* < 0.05).

We next explored the involvement of CatS in splenic IL-6 production after exposure to PgLPS, as IL-6 is an essential factor for Th17 differentiation. The mean protein level of CatS in the spleen of wild-type mice was significantly higher than that in the saline-exposed mice, paralleling the increase in the expression of IL-6 (**Figures 3A–C**). However, the mean protein level of IL-6 was not increased in the spleen of the *CatS^-/-^* mice after exposure to PgLPS (**Figures 3A,C**). Furthermore, the immunoreactivities for CatS paralleled those of IL-6 in splenic CD11c^+^ cells but not in CD4^+^ cells in wild-type mice (**Figures 3D–F**), and the mean percentage of splenic CD11c ^+^ cells expressing CatS and IL-6 was 64% ± 2.5% (*n* = 6) and 40% ± 2.1% (*n* = 6), respectively, after exposure to PgLPS. These results strongly suggest that CatS is involved in IL-6 production by CD11c^+^ DCs in the splenic MZ after systemic exposure to PgLPS.

### CatS Regulates the Production of IL-6 by Splenic CD11c^+^ DCs by Activating PAR2/PI3K/Akt Signaling after Exposure to PgLPS

**FIGURE 4:**
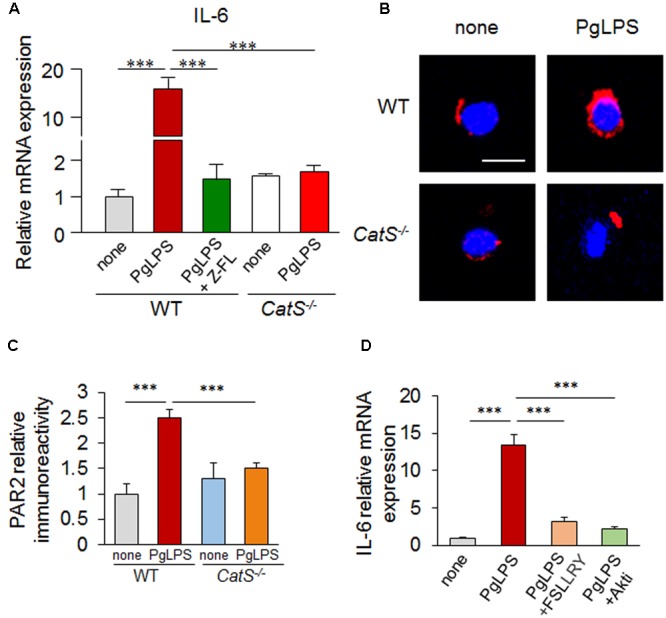
CatS regulates the IL-6 production by splenic CD11c^+^ DCs via the activation of PAR2/PI3K/Akt signaling after exposure to PgLPS for 24 h. **(A)** The mRNA expression of IL6 in the isolated primary splenic CD11c^+^ cells. The increased expression of IL-6 by PgLPS can be suppressed by either the genetic deletion or pharmacological inhibition of CatS. Each column and vertical bar represent the mean ± SEM of four independent experiments (*n* = 4). Asterisks indicate a statistically significant difference between the values (^∗∗∗^*p* < 0.001). **(B)** The immunofluorescent CLSM images of PAR2 (red) in the CD11c^+^ cells isolated from wild-type and *CatS^-/-^* mouse spleens challenged with PgLPS. Scale bar, 15 μm. **(C)** The quantitative analyses of PAR2 in the CLSM images shown in **(B)**. Each column and vertical bar represent the mean ± SEM of four independent experiments (n = 4). Asterisks indicate a statistically significant difference between the values (^∗∗∗^*p* < 0.001). **(D)** The changes in the levels of IL-6 mRNA in the isolated CD11c^+^ cells in the presence of PgLPS or on pre-treatment with FSLLRY (PAR2 antagonist) and Akti (Akt inhibitor) for 1 h. Each column and vertical bar represent the mean ± SEM of four independent experiments (*n* = 4). Asterisks indicate a statistically significant difference between the values (^∗∗∗^*p* < 0.001).

To further confirm the involvement of CatS in the IL-6 production by splenic DCs after exposure to PgLPS, we next directly exposed isolated splenic CD11c^+^ primary DCs from wild-type and *CatS^-/-^* mice to PgLPS. The mean mRNA expression of IL-6 was dramatically higher in the CD11c^+^ DCs from wild-type mice (17-fold) after exposure for 24 h than in the non-PgLPS-exposed primary splenic CD11c^+^ DCs, and the PgLPS-enhanced mRNA expression of IL-6 in CD11c^+^ DCs was completely blocked by pre-treatment with by Z-FL, a specific CatS inhibitor (**Figure 4A**). Furthermore, the mRNA expression of IL-6 in the isolated primary CD11c^+^ DCs from *CatS^-/-^* mice was not increased after exposure to PgLPS for 24 h (**Figure 4A**). These observations confirmed that pharmacological blockage or genetic deletion of CatS completely blocked the PgLPS-induced IL-6 by splenic CD11c^+^ DCs, strongly suggesting that CatS plays a critical role in IL-6 production by splenic CD11c^+^ DCs after PgLPS exposure.

We next focused on the PAR2 activation and signaling pathways after exposure to PgLPS using the isolated CD11c^+^ DCs, because the CatS-cleaved PAR2 activation is related to the IL-6 expression (Cattaruzza et al., 2011; Viode et al., 2014). The immunoreactivities for PAR2 were significantly higher in the CD11c^+^ DCs from wild-type mice than in the non-PgLPS-exposed isolated CD11c^+^ DCs, but not in those from *CatS^-/-^* mice, after exposure to PgLPS for 24 h (**Figures 4B,C**). In addition, the PgLPS-induced increase in the mRNA expression of IL-6 in CD11c^+^ DCs from wild-type mice was completely abolished by FSLLRY-NH_2_, an antagonist of PAR2 (**Figure 4D**). These results indicate that CatS-activated PAR2 is required for IL-6 expression by splenic DCs after exposure to PgLPS. Furthermore, the PgLPS-enhanced mRNA expression of IL-6 in isolated CD11c^+^ DCs from wild-type mice was significantly inhibited by Akti, a specific inhibitor of Akt (**Figure 4D**). Taken together, these results indicate that CatS is involved in the IL-6 production by splenic CD11c^+^ DCs via the PAR2/PI3K/Akt signaling pathways after exposure to PgLPS.

### PgLPS-Induced IL-6 by DCs Is Required for Splenic Th17 Differentiation

**FIGURE 5:**
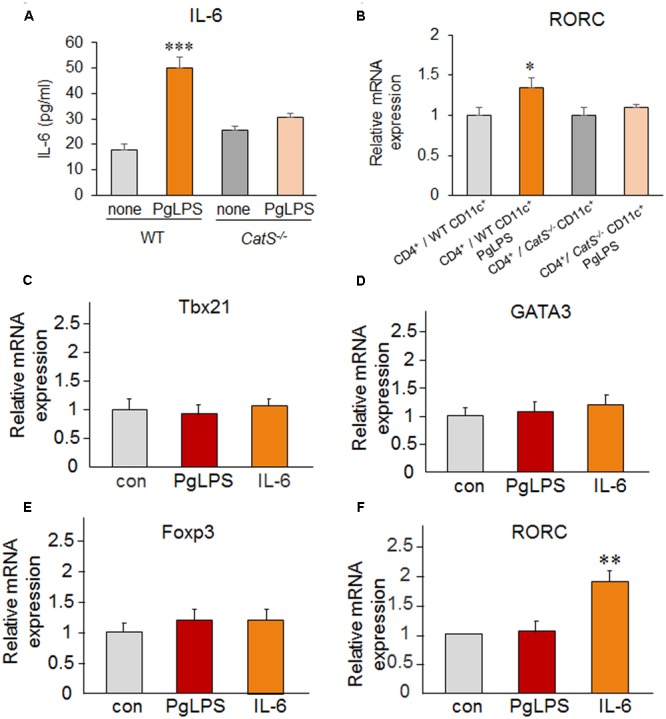
PgLPS-induced IL-6 by DCs is required for splenic Th17 differentiation. **(A)** The mean amount of IL-6 secreted from WT and *CatS^-/-^* CD11c^+^ cells after exposure to PgLPS for 24 h (1 μg/ml). Each column and vertical bar represent the mean ± SEM of four independent experiments (n = 4). Asterisks indicate a statistically significant difference between the values (^∗∗∗^*p* < 0.001). **(B)** The change in the mRNA of RORC in the CD4^+^ cells co-cultured with WT or *CatS^-/-^* CD11c^+^ cells. Each column and vertical bar represent the mean ± SEM of four independent experiments (n = 4). Asterisks indicate a statistically significant difference between the values (^∗^*p* < 0.05). **(C–F)** The change in the mRNA expression of Tbx21 **(C)**, GATA3 **(D)**, Foxp3 **(E)**, and RORC **(F)** in the isolated splenic primary CD4^+^ T cells from wild-type mice in the presence of PgLPS (1 μg/ml) and IL-6 (10 ng/ml) for 24 h. Each column and vertical bar represent the mean ± SEM of four independent experiments (*n* = 4). Asterisks indicate a statistically significant difference between the values (^∗∗^*p* < 0.01).

**FIGURE 6:**
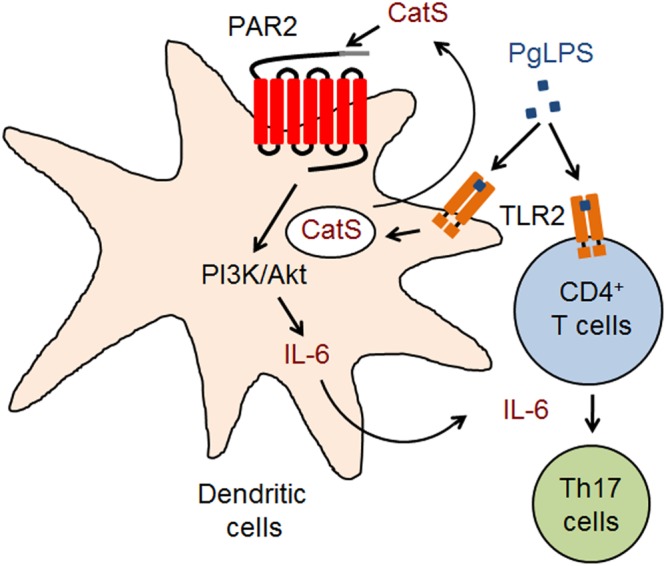
A schematic representation of the critical role of CatS in splenic Th17 differentiation after systemic exposure to PgLPS. CD11c^+^ cells are activated to produce CatS for activating PAR2-dependent IL-6 through the PI3K/Akt pathway, which induces the TLR2-activated CD4^+^ T cells into Th17 cells after exposure to PgLPS.

To further confirm the involvement of DC-derived IL-6 in subtype differentiation of splenic CD4^+^ T cells, we examined the secretion of IL-6 from the cultured CD11c^+^ cells and its effects on the genes specific for CD4^+^ T cell subtype differentiation. The protein level of IL-6 secreted from WT CD11c^+^ cells but not from *CatS^-/-^* CD11c^+^ cells was significantly increased after exposure to PgLPS for 24 h compared with non-PgLPS- exposed cells (**Figure 5A**). Furthermore, a significant increase in the IL-6 secretion was also detected from the co-cultured media of the WT CD4^+^ T cells with WT CD11c^+^ cells but not with *CatS^-/-^* CD11c^+^ cells (data not shown). In contrast, a significant increase in the mRNA of RORC (the specific gene for Th17 differentiation) was detected in the CD4^+^ cells co-cultured with WT CD11c^+^ cells, but not in the CD4^+^ cells co-cultured with *CatS^-/-^* CD11c^+^ cells after exposure PgLPS for 24 h (**Figure 5B**). To further confirm the direct effect of IL-6 on the differentiation of splenic CD4^+^ cells, we treated CD4^+^ cells with recombinant IL-6. The mean mRNA expressions of Tbx21 (the specific gene for Th1 differentiation), GATA3 (the specific gene for Th2 differentiation), Foxp3 (the specific gene for Treg differentiation) and RORC were not significantly increased in the primary CD4^+^ T cells from the spleens of wild-type mice after exposure to PgLPS which compared with those after exposure to saline (**Figures 5C–F**), suggesting that subtype differentiation of splenic CD4^+^ T cells was not directly induced by PgLPS. Of note, the mean mRNA expression of RORC but not that of Tbx21, GATA3 or Foxp3 in the splenic primary CD4^+^ T cells was significantly increased at 24 h after treatment with IL-6 (**Figures 5C–F**), demonstrating that DC-produced IL-6 was necessary for Th17 differentiation from splenic CD4^+^ T cells after exposure to PgLPS. The critical roles of CatS in regulating splenic DCs-dependent Th17 cell differentiation after systemic exposure to PgLPS are summarized in **Figure 6**.

## Discussion

The major finding of the present study was our clarification of the critical role of CatS in driving DC-dependent Th17 differentiation during systemic exposure to PgLPS (summarized in **Figure 6**). To our knowledge, this is the first study to demonstrate the involvement of CatS in splenic immune responses during TLR2 signaling, thereby adding valuable information regarding the mechanisms of the persistence of the immune response/inflammation during systemic exposure to periodontal bacterial components.

In the present study, we focused on the response of spleen, the largest SLO during systemic exposure to PgLPS (Bronte and Pittet, 2013; Tarantino et al., 2013), because the spleen is susceptible to infections (Tarantino et al., 2011). Systemic exposure to PgLPS in DBA/2 mice for 7 consecutive days mimicked approximately 10 months’ exposure to periodontal bacteria components in the human bloodstream, as the average lifespan of DBA/2 mice is 1/40 that of humans (Festing and Blackmore, 1971). To ensure a biologic effect, a high dose of PgLPS (5 mg/kg) was used in the present study, the same dose at which other TLR2 agonists have been used in mice (Eklind et al., 2004; Du et al., 2011). No mortality or other signs of morbidity was induced with this dose of PgLPS. Furthermore, no significant reduction in body weights (**Figure 1D**) or the induction of lethargy (data not shown) was detected in the PgLPS-exposed mice during the experimental period, suggesting that the dose of PgLPS used did not induce sickness. These findings, taken together with our recent results showing that systemic exposure to PgLPS (1 mg/kg) in mice for 5 consecutive weeks did not induce sickness (Wu et al. unpublished observation), support the notion that TLR2 agonists have less potency in mammals than TLR4 agonists such as *E. coli* LPS (Hirschfeld et al., 2001; Du et al., 2011; Anderson et al., 2015).

We noted particularly accumulations of CD11c^+^ and CD4^+^ T cells in the MZ after systemic exposure to PgLPS (**Figures 1, 2**), suggesting that DCs and CD4^+^ T cells in the MZ sensitively responded to periodontal bacteria/bacterial components in the bloodstream because of their close interfacing (Ruddle and Akirav, 2009; Tarantino et al., 2011; Zhao et al., 2015) and the PgLPS-accumulated CD11c^+^ and CD4^+^ T cells facilitate their effective interaction (Bajenoff et al., 2008; Bronte and Pittet, 2013). Of note, the PgLPS-induced accumulation of CD11c^+^ and CD4^+^ T cells in the MZ of wild-type mice was strongly inhibited in *CatS^-/-^* mice (**Figures 1, 2**), indicating that CatS is involved in the splenic DC/CD4^+^ T cell interactions after exposure to PgLPS (Gravina et al., 2013). However, whether or not CatS is involved in the proliferation of splenic DCs/CD4^+^ T cells in the MZ must be further investigated in the future.

CD11c^+^ DCs initiate naïve T cell responses on their activation (Hertz et al., 2001). In the present study, the TLR2 expression was significantly increased in both CD11c^+^ DCs and CD4^+^ T cells after systemic exposure to PgLPS, a TLR2 ligand (Liu et al., 2013; Li et al., 2016), supporting the notion that DCs and CD4^+^ T cells can be activated during TLR2 signaling (Hertz et al., 2001; Biswas et al., 2009; Pene et al., 2009; Reynolds et al., 2010; Owens et al., 2012). The PgLPS-enhanced TLR2 expression in splenic CD11c^+^ and CD4^+^ T cells was not inhibited in *CatS^-/-^* mice (**Figures 1, 2**), suggesting that the effects of CatS are elicited downstream of TLR2 activation.

Dendritic cells direct naïve CD4^+^ T cells into functionally subtypes, such as Th1 (IFN-γ^+^ CD4^+^), Th2 (IL-4^+^ CD4^+)^, Th17 (Th17^+^ CD4^+^), or Treg (TGFβ^+^ CD4^+^) cells (McGuirk and Mills, 2002; Harrington et al., 2005). In the present study, we noted a dramatic accumulation of Th17^+^ CD4 T^+^ cells near the MZ in PgLPS-treated wild-type mice but not *CatS^-/-^* mice (**Figure 2**), suggesting that CatS is specifically involved in the PgLPS-induced differentiation of Th17 cells. PgLPS promoted splenic Th17 cell differentiation, which agreed with the *P. gingivalis* infections-induced Th17 responses (Shabgah et al., 2014). CatS is active at neutral pH, exerting extracellular biological activity (Storm van’s Gravesande et al., 2002), unlike other Cathepsins, which are generally activated in the lysosomal acidic surroundings (Sun et al., 2012). Functionally distinguished from CatB, which is involving in IL-1β production (Sun et al., 2012; Wu et al., 2013), CatS mainly controls MHC class II antigen-presenting cells, including DCs (Shi et al., 1999; Riese et al., 2001; Nakanishi, 2003; Zhang et al., 2014). We also found that CatS is involved in PgLPS-induced splenic Th17 cell differentiation (**Figures 2, 5**).

Activated CD4^+^ T cell subset differentiation is strictly controlled by cytokine environments (Dong, 2008; Yamane and Paul, 2012), and IL-6 is necessary for DC-dependent Th17 cell differentiation and IL-17 production (Ivanov et al., 2006; Yang et al., 2008). Our data showed that IL-6 was markedly induced in splenic CD11c^+^ cells of MZ after systemic exposure to PgLPS. This finding, taken together with the significantly increase of the production of IL-6 in the isolated splenic CD11c^+^ cells after direct exposure to PgLPS (1 μg/ml) (**Figures 3, 4**), suggests that the TLR2-dependent IL-6 production by DCs is necessary to drive Th17 cell differentiation (Fedele et al., 2008; Pene et al., 2009). Furthermore, the PgLPS-induced IL-6 from DCs, but not PgLPS itself, significantly increased the mRNA expression of RORK, but not that of Tbx21, GATA3 or Foxp3 in the isolated splenic CD4^+^ T cells, indicating that PgLPS-induced IL-6 from DCs drives the splenic Th17 differentiation at the transcription level (Zhou et al., 2005; Acosta-Rodriguez et al., 2007; Mucida et al., 2007; Dong, 2008). Concordantly, the PgLPS-induced increase in the IL-6 expression was completely blocked by genetic CatS deficiency and administration of the specific CatS inhibitor, Z-FL (**Figures 3, 4**), indicating the critical role of CatS in regulating the PgLPS-induced production of IL-6 by DCs. TGFβ plays a synergistic role with IL-6 in Th17 cell differentiation (Mangan et al., 2006; Acosta-Rodriguez et al., 2007; Bettelli et al., 2008). The TGFβ expression was not increased after systemic exposure to PgLPS (**Figures 2, 5**), suggesting that PgLPS-induced splenic Th17 cell differentiation may not be related to TGFβ. However, we could not exclude the involvement of monocyte/macrophages in the activated CD4^+^ T cell subset differentiation because of the cytokine production. Of note, the expression of CatS in splenic CD11b^+^ monocyte/macrophages (40% corresponded) was not higher than that in CD11c^+^ DCs in the splenic MZ (64% corresponded) after systemic exposure to PgLPS, supporting the notion that the TLR response in monocytes/macrophages can be controlled by DCs (Gravina et al., 2013).

PAR2, which is expressed on DCs (Fields et al., 2003; Mohamadzadeh et al., 2004), has been identified as a novel upstream up-regulator of IL-6 for immune response (Shpacovitch et al., 2008; Lewkowich et al., 2011; Crilly et al., 2012). In the present study, PAR2 levels were markedly elevated in isolated splenic CD11c^+^ DCs by PgLPS, and the PgLPS-induced increase in the IL-6 expression was completely inhibited by FSLLRY-NH_2_, an antagonist of PAR2, indicating that the PgLPS-induced IL-6 production was PAR2 activation-dependent.

PAR2 is activated followed its proteolytic cleavage of extracellular N-terminal domains by serine proteases (Mohamadzadeh et al., 2004), and CatS cleaves PAR2’s N-terminal domains (Elmariah et al., 2014; Zhao et al., 2014). In the present study, the PgLPS-induced PAR2 activation in the isolated splenic DCs from wild-type mice was completely inhibited in *CatS^-/-^* mice (**Figure 5**), indicating that CatS is involved in PAR2 activation during inflammation (Cattaruzza et al., 2011; Viode et al., 2014). CatS is active at a neutral pH, consistent with its extracellular biological activity (Storm van’s Gravesande et al., 2002), and the PgLPS-induced increase in the IL-6 mRNA expression in splenic DCs from wild-type mice was significantly inhibited by Akti, a specific inhibitor Akt kinase, which is consistent with the findings from previous reports showing CatS-activated PAR2 signaling through the PI3K/Akt pathway in human glioblastoma cells (Dutra-Oliveira et al., 2012). Taken together, these findings suggest that CatS-activated PAR2 coupling to the PI3K/Akt signaling pathways in splenic DCs may cause IL-6-dependent splenic Th17 cell differentiation after exposure to PgLPS (**Figure 6**).

IL-6 and IL-17 play synergistic roles in enhancing inflammation as follows: First, IL-6 and IL-17 induce inflammation directly or indirectly by enhancing other inflammatory mediators (Ito, 2003; Carey et al., 2008). Second, Th17 cells driven by IL-6 can be recruited to the remote organs. Indeed, Th17 T cells infiltrated into brain of AD patients (Lambracht-Washington et al., 2011; Saresella et al., 2012). Third, IL-6 and IL-17 up-regulate each other (Muranski and Restifo, 2013). Therefore, CatS-dependent splenic IL-6/andIL-17 is considered as the resources for inducing and/or lasting immune responses/inflammation. Therefore, splenic MZ, may be an effective pharmacological target for regulating the immune response/inflammation in the whole body.

## Conclusion

We found that CatS plays a critical role in driving splenic CD11c^+^ DC-dependent Th17 differentiation through the upregulation of IL-6 by activating PAR2 after exposure to components of periodontal bacteria. As such, CatS-specific inhibitors may be useful for alleviating periodontitis-related immune response/inflammation.

## Author Contributions

MD, ZW, and JN contributed equally to this work. ZW and JN designed the experiments. ZW and HN supervised the project. MD and JN collected the data mainly. XZ, YL, and XY collected the data. MD, JN, IT, XZ, YL, and XY analyzed the data. ZW and HN wrote the manuscript. IT provided advices for manuscript.

## Conflict of Interest Statement

The authors declare that the research was conducted in the absence of any commercial or financial relationships that could be construed as a potential conflict of interest.
